# GLP-1 receptor and Mitochondria-ER Contact Sites: an emerging mechanism of metabolic regulation

**DOI:** 10.3389/fphys.2026.1826699

**Published:** 2026-04-28

**Authors:** Pablo Cruz, Andrea Puebla-Huerta, Mayarling F. Troncoso, Eduardo Silva-Pavez, Yessia Hidalgo-Fadic, Ulises Ahumada-Castro

**Affiliations:** 1 Departamento de Tecnología Médica, Facultad de Medicina, Universidad de Chile, Santiago, Chile; 2 Centros de Investigación Clínica Avanzada, Hospital Clínico Universidad de Chile, Universidad de Chile, Santiago, Chile; 3 Advanced Center for Chronic Diseases (ACCDiS), Facultad de Ciencias Químicas y Farmacéuticas & Facultad de Medicina, Universidad de Chile, Santiago, Chile; 4 Facultad de Odontología, Universidad San Sebastián, Santiago, Chile; 5 IMPACT, Center of Interventional Medicine for Precision and Advanced Cellular Therapy, Santiago, Chile; 6 Laboratory of Nano-Regenerative Medicine, Biomedical Research and Innovation Center (CIIB), Universidad de los Andes, Santiago, Chile

**Keywords:** GLP-1 receptor (GLP-1R), metabolic dysfunction, Mitochondria-ER Contact Sites (MERCS), signalosomes, GLP1 receptor agonist (GLP1-RAs)

## Abstract

The uncoupling of Mitochondria-ER Contact Sites (MERCS) represents a hallmark of metabolic dysfunction in obesity and type 2 diabetes. These are dynamic interfaces that play essential roles in coordinating ion signaling and lipid exchange to maintain cellular homeostasis. Persistent organelle stress in chronic disease impairs these pathways, driving systemic hormone resistance and metabolic failure. A paradigm shifts in glucagon-like peptide-1 receptor (GLP-1R) signaling from diffuse events to spatiotemporally organized signalosomes offer insightful mechanisms into these conditions. The localization of internalized GLP-1Rs at the Mitochondria-ER interface supports a contactomics framework for understanding bioenergetic restoration. Stabilizing inter-organellar connectivity represents a novel frontier for next-generation metabolic therapies.

## 1 Introduction

It is well-documented that eukaryotic organelles do not operate as isolated metabolic islands but instead function within a highly integrated network facilitated by specialized Membrane Contact Sites (MCS) (Prinz et al., 2020; Scorrano et al., 2019). These structural interfaces are defined as regions in which organelle membranes are brought into proximity, typically within 10–80 nm, thereby enabling direct communication and non-vesicular exchange of ions, lipids, and signaling molecules without membrane fusion (Cali et al., 2025). Evidence has established that MCS are essential for maintaining cellular homeostasis, serving as spatiotemporal hubs that coordinate diverse processes, ranging from lipid biogenesis to the regulation of second messengers (Voeltz et al., 2024). Among these junctions, Mitochondria-Endoplasmic Reticulum (ER) Contact Sites (MERCS) have emerged as the primary archetype of this communication system, serving as the metabolic core of the cell by matching energy production with physiological demand. Chronic disruption and uncoupling of these interfaces are increasingly recognized as a hallmark of metabolic disorders, including obesity, type 2 diabetes (T2DM), and metabolic dysfunction-associated steatohepatitis (MASH) (Beaulant et al., 2022; Rieusset, 2018). The glucagon-like peptide-1 (GLP-1) signaling axis has driven a paradigm shift in the management of these metabolic conditions, emerging as a master regulator of cellular homeostasis; however, its profound molecular intersection with MERCS remains a critical yet largely underestimated frontier (Kanwal, 2024; Olukorode et al., 2024; Son and Lim, 2024). This review synthesizes the critical milestones that position MERCS as essential signaling platforms for GLP-1 receptor (GLP-1R) function in both health and disease. Finally, we address outstanding questions about how inter-organellar connectivity specifies GLP-1 signaling outcomes and evaluate its potential as a framework for next-generation metabolic therapies.

## 2 Mitochondria-ER Contact Sites communication as the metabolic core in health and diseases

Characterized as dynamic signaling platforms, MERCS are maintained at 10 to 50 nm (Giacomello and Pellegrini, 2016). The extent of these contacts is remarkably plastic; in healthy mammalian cells, between 5% and 20% of the total mitochondrial surface area is in direct apposition with the ER network (Rizzuto et al., 1998). The architectural integrity of these structural juxtapositions is governed by molecular tethers, as MFN2 (Naon et al., 2016), which forms homotypic or heterotypic dimers between the outer mitochondrial membrane and the ER; VAPB-PTPIP51 complex (Gomez-Suaga et al., 2017), Fis1-BAP31 complex (Iwasawa et al., 2011), and the recently identified PDZD8-FKBP8 tether, which regulates mitochondrial complexity and morphology (Nakamura et al., 2025; Obara et al., 2024).

Beyond their structural features, MERCS are established as essential regulatory hubs for a vast array of cellular phenomena. Evidence indicates that these interfaces coordinate Ca^2+^ signaling microdomains, non-vesicular lipid transfer, and mitochondrial bioenergetics to maintain proteostasis, among other functions (Carreras-Sureda et al., 2022; Dematteis et al., 2024; Giacomello and Pellegrini, 2016; Sassano et al., 2022). The transfer of Ca^2+^ from the ER to the mitochondria is the most characterized functional event at MERCS. The architecture of these sites creates “quasi-synaptic” microdomains in which local concentrations can reach 10-100 µM, thereby overcoming the low affinity (roughly K*d* approx 20-30 µM) of the mitochondrial calcium uniporter (mtCU) (Csordas et al., 1999, 2010; Eisner et al., 2013; Fieni et al., 2012; Kamer et al., 2017; Marchi and Pinton, 2014; Rapizzi et al., 2002; Rizzuto et al., 1998). The core machinery of this axis is the IP3R-GRP75-VDAC1 tripartite complex, where GRP75 (Szabadkai et al., 2006) acts as a linker protein that physically bridges the IP3R on the ER (Bartok et al., 2019) to the VDAC1 channel on the mitochondria (Rapizzi et al., 2002), funneling ions directly into the mitochondrial matrix (Szabadkai et al., 2006). Ultrastructural analysis using high-resolution electron microscopy has identified approximately 20 nm as the biophysical optimum for efficient transfer (Csordas et al., 2006; Dematteis et al., 2024). At this ~20 nm gap, the concentration of released Ca^2+^ creates microdomains that facilitate the activation of mtCU (Csordas et al., 2010). Once Ca^2+^ enters the mitochondrial matrix, it acts as a critical regulator of the Krebs cycle. Specifically, it stimulates the activity of three rate-limiting dehydrogenases: pyruvate dehydrogenase (PDH), isocitrate dehydrogenase (IDH), and -ketoglutarate dehydrogenase (α-KGDH) (Denton, 2009). This stimulation enhances the supply of NADH (Robb-Gaspers et al., 1998) to the electron transport chain, thereby sustaining oxidative phosphorylation and ATP synthesis (Tarasov et al., 2012). During metabolic stress, the Sigma-1 receptor (S1R) plays a pivotal role in stabilizing IP3Rs at the contact site, preventing their degradation and maintaining the bioenergetic flow required for cellular survival (Hayashi and Su, 2007; Mori et al., 2013).

Another interesting MERCS function is related to lipid synthesis. Because mitochondria are functionally isolated from canonical vesicular pathways, the delivery of phospholipids is dependent on non-vesicular transport at MERCS (Mori et al., 2013; Petrungaro and Kornmann, 2019; Shiao et al., 1998). The most prominent metabolic route is the conversion of phosphatidylserine (PS) to phosphatidylethanolamine (PE). PS synthesized in the ER is transported to the mitochondria, where it is converted into PE by the enzyme phosphatidylserine decarboxylase (PISD) (Kang et al., 2008; Kannan et al., 2017; Luan et al., 2025; Petrungaro and Kornmann, 2019; Tamura et al., 2012). This exchange is facilitated by lipid transfer proteins such as ORP5 and ORP8, which interact with the mitochondrial intermembrane space bridge (MIB) and cristae junction organizing system (MICOS) complexes (Galmes et al., 2016; Guyard et al., 2022; Monteiro-Cardoso et al., 2022). Furthermore, bridge-like transporters such as VPS13D and MIGA2 have emerged as essential components. VPS13D forms a rod-like conduit with a hydrophobic groove through which multiple lipids can slide simultaneously (Guillen-Samander et al., 2021; Neuman et al., 2022; Swan, 2025), while MIGA2 integrates mitochondrial metabolism with cellular fat storage by serving as a bridge to lipid droplets transfer (Freyre et al., 2019; Hong et al., 2022; Kim et al., 2022; Xu et al., 2020).

MERCS also integrates sensors of the unfolded protein response (UPR), including PERK and IRE1α (Bassot et al., 2023; Carreras-Sureda et al., 2019, 2022; Verfaillie et al., 2012). A critical emerging mechanism in proteostasis is the recruitment of TANK-binding kinase 1 (TBK1) to the MERCS interface. Evidence established that a MERCS-specific E3 ubiquitin ligase, autocrine motility factor receptor (AMFR), ubiquitinates nascent proteins to activate TBK1 at the contact site (Watanabe et al., 2023). This AMFR-S1R-TBK1 linkage provides a direct molecular link between protein quality control and organellar signaling, although its full significance in human metabolic disease remains under investigation.

As briefly shown, the dynamic reorganization of MERCS directly determines cell fate in response to metabolic stress (Hayashi and Su, 2007; Hoffmann et al., 2026; Nakamura et al., 2025; Obara et al., 2024; Sassano et al., 2025; Verfaillie et al., 2012). Chronic disruption of their architecture is a primary driver of metabolic alterations (Theurey and Rieusset, 2017; Tubbs et al., 2018). In the skeletal muscle of obese and diabetic patients, a significant reduction in MFN2 expression has been correlated with impaired glucose oxidation and insulin resistance (Bach et al., 2005). This decoupling of the ER and mitochondria disrupts the flux required to sustain mitochondrial ATP production (Rizzuto et al., 2009). Conversely, evidence indicates that early-stage hepatic insulin resistance in mouse models is associated with a chronic enrichment of MERCS. This excessive connectivity leads to chronic mitochondrial Ca^2+^ overload, triggering reactive oxygen species (ROS) production and hepatic UPR activation (Arruda et al., 2014; Jelenik et al., 2017). Researchers speculate that this discrepancy indicates a nonlinear trajectory in which early adaptive enrichment eventually gives way to pathological decoupling as the disease progresses. In MASH, the breakdown of the PS-PE lipid transport route is critical (Anari et al., 2024), and a deficiency in hepatic MFN2 results in reduced PS transfer, which induces lipotoxic ER stress and bioenergetic collapse. This structural failure impairs the liver’s ability to process lipid flux, thereby driving the progression of steatosis and fibrosis (Hernandez-Alvarez et al., 2019).

Notably, current pharmacological strategies for metabolic disorders have identified the normalization of MERCS connectivity as a pivotal therapeutic target (Bui et al., 2026; Magalhaes Rebelo et al., 2020; Yang et al., 2021). Metformin, the cornerstone of T2DM therapy, activates AMP-activated protein kinase (AMPK), which has been shown to normalize SR/ER-mitochondria interactions (Angebault et al., 2020). Under conditions of hyperglycemia, AMPK activation suppresses the aberrant formation of contacts that leads to mitochondrial Ca^2+^ overload (Wu et al., 2019). Furthermore, thiazolidinediones, such as pioglitazone, affect the interface by binding to NAF-1 (also known as mitoNEET) (Paddock et al., 2007). Pioglitazone stabilizes the clusters of this redox-active iron-sulfur protein at the MERCS, preventing deleterious ultrastructural changes and protecting against the lipid peroxidation that drives hepatic steatosis (Paddock et al., 2007; Tomita et al., 2004).

While classical pharmacological tools like metformin and pioglitazone underscore the therapeutic value of MERCS stabilization, these agents primarily function as intracellular sensors and modulators (Angebault et al., 2020; Paddock et al., 2007). However, metabolic disorders are fundamentally systemic diseases driven by impaired endocrine crosstalk (Theurey and Rieusset, 2017; Tubbs et al., 2018). Therefore, identifying how systemic hormonal signals coordinate with local organelle architecture remains a critical gap in our understanding of these pathologies. In this context, the glucagon-like peptide-1 (GLP-1) receptor system has emerged as a paradigm-shifting model (Austin and Tomas, 2026). Beyond its classical endocrine role, the GLP-1 axis offers a compelling window into the regulatory mechanisms that govern inter-organellar communication. By organizing into spatiotemporal signalosomes directly at the MERCS, the GLP-1 receptor serves as a pivotal molecular link between whole-body glucose regulation and the precise maintenance of intracellular structural integrity (Anton et al., 2022; Austin et al., 2025).

## 3 GLP-1 receptor signaling and GLP-1RA in metabolic disorders

The global prevalence of metabolic dysfunction, encompassing obesity and T2DM, has reached pandemic proportions (Chew et al., 2023), characterized by a complex interplay of insulin resistance (Barber et al., 2021), low-grade systemic inflammation (Rohm et al., 2022), and organelle dysfunction (Arruda and Hotamisligil, 2015; Arruda et al., 2014; Chang et al., 2015; Lemmer et al., 2021). Within this multifaceted pathological landscape, the GLP-1 system has emerged as a cornerstone of modern metabolic pharmacology, transcending its initial role as a simple glycemia-regulating incretin (Kong et al., 2026; Muller et al., 2019; Tanday et al., 2022). Its therapeutic prominence is rooted in its ability to simultaneously address systemic hyperglycemia, central appetite regulation, and the chronic organelle stress that drives tissue-specific failure (Pereira et al., 2021; Zheng et al., 2024). The biological actions of GLP-1 across a wide range of tissues, including the pancreas, brain, cardiovascular system, and liver, are mediated through the GLP-1R, a class B1 G protein-coupled receptor (GPCR) (Zheng et al., 2024). Recent high-resolution structural studies, primarily using cryogenic electron microscopy (cryo-EM), have established a unique “two-domain” binding mechanism of the GLP-1R. Upon activation, the receptor primarily couples to the stimulatory G_s_, stabilized by specific contacts with residues such as R176^2.46b^ and Y402^7.57b^ (Song et al., 2017; Zhang et al., 2017; Zhou et al., 2025). This coupling stimulates adenylate cyclase (AC), particularly the AC8 isoform in pancreatic β-cells, resulting in a rapid increase in cyclic adenosine monophosphate (cAMP) (Roger et al., 2011). The subsequent activation of protein kinase A (PKA) and exchange protein directly activated by cAMP 2 (Epac2) is also established (Holz, 2004; Leech et al., 2010; Meloni et al., 2013). Evidence demonstrates that PKA phosphorylates exocytotic machinery and transcription factors like CREB to enhance insulin biosynthesis and secretion (Dalle et al., 2011; Jhala et al., 2003; Wu et al., 2015). Simultaneously, the Epac2 pathway is known to sensitize β-cells to glucose by lowering the ATP threshold required for K_ATP_ channel closure (Kang et al., 2006, 2008; Yosida et al., 2014).

**Figure 1:**
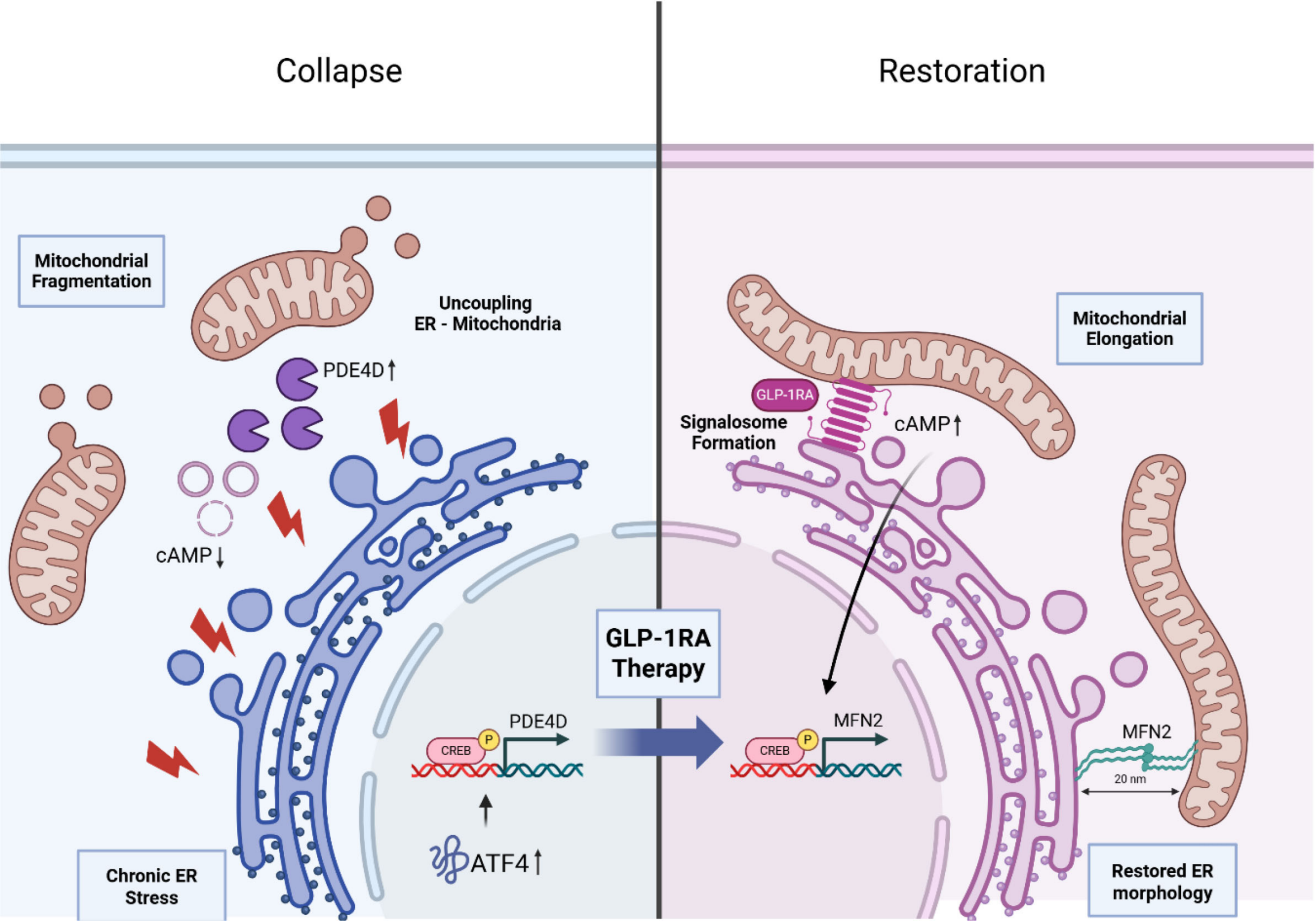
The molecular mechanisms of incretin resistance versus pharmacological normalization at Mitochondria-ER Contact Sites. Left (Collapse): This panel illustrates the “incretin resistance cycle” prevalent in metabolic dysfunction. Chronic ER stress triggers the nuclear accumulation of ATF4, which subsequently induces the transcription of phosphodiesterase 4D (PDE4D). The resulting upregulation of PDE4D accelerates intracellular cAMP degradation, blunting cAMP-mediated signaling fidelity. This disrupted signaling environment results in the structural uncoupling of mitochondria from the ER interface and mitochondrial fragmentation, which are hallmarks of metabolic inflexibility. Right (Restoration): This panel depicts cellular recovery following GLP-1 Receptor Agonist (GLP-1RA) therapy. The pharmacological agonist drives the organization of the GLP-1R into specialized “signalosomes” localized at the MERCS, accompanied by a restoration of ER morphology. Within this nanodomain, localized cAMP accumulation promotes the CREB-dependent transcriptional upregulation of the tethering protein MFN2. This leads to mitochondrial elongation and the re-establishment of functional 20 nm coupling distances between the ER and mitochondria, restoring cellular homeostasis. Created with BioRender.com.

In chronic metabolic states like obesity and T2DM, the GLP-1R signaling axis undergoes significant alterations that drive incretin resistance and impaired glucose control (Rabbani and Thornalley, 2024; Tzeravini et al., 2026). Evidence indicates that chronic hyperglycemia and elevated free fatty acids (FFAs) levels trigger persistent ER stress, specifically by activating the PERK-eIF2α-ATF4 pathway (Back and Kaufman, 2012; Lemmer et al., 2021). Research has established that ATF4 directly induces the transcription of PDE4D, a phosphodiesterase that degrades intracellular cAMP, thereby blunting the signaling fidelity of the GLP-1R (Lee et al., 2023). This causal link is supported by robust molecular evidence from models such as the *db/db* mouse, in which nuclear localization of ATF4 in β-cells correlates with diminished insulinotropic responses and accelerated mass loss (Lee et al., 2023) (**Figure 1**, left).

In these diseases, metabolic dysfunction is further compounded by lipotoxicity in non-adipose tissues; for example, in MASH, the abnormal accumulation of lipotoxic species, such as ceramides and diacylglycerols (DAGs), triggers inflammatory signaling that antagonizes receptor-mediated protection (Steinberg et al., 2025). FFAs induce oxidative stress by generating ROS, which promote lipid peroxidation and activate cell death pathways, thereby exacerbating the vicious cycle between mitochondrial failure and ER stress (Carli et al., 2024; Khoi et al., 2024; Lytrivi et al., 2020). Furthermore, unscheduled glycolysis and glycolytic overload in enteroendocrine cells and neurons are speculated to exacerbate impairment of the incretin effect, creating a systemic landscape of resistance that links organelle failure to clinical metabolic decline (Meier and Nauck, 2010; Nauck, 2009; Rabbani and Thornalley, 2023, 2024). These mechanisms indicate that decrements in GLP-1R efficacy are not merely the result of receptor downregulation but are rooted in a fundamental disruption of the intracellular environment, driven by organelle stress and lipotoxic signaling. Counteracting this complex landscape, this necessitated the development of synthetic agonists that provide a robust and persistent stimulus, thereby overcoming the pharmacokinetic limitations of the native hormone. Because the transient nature of endogenous GLP-1 is insufficient to override the intracellular “resistance” seen in chronic disease, pharmacological innovation has focused on engineering molecules that can sustain the receptor’s protective signalosomes over extended periods (**Figure 1**, right).

The pharmacological development of GLP-1R agonists (GLP-1RAs) has centered on extending the half-life of the native hormone, which is certain to be degraded by DPP-4 within two minutes (Gong et al., 2024; He et al., 2024; Muller et al., 2019). The first generation of drugs, such as exenatide, utilized a synthetic version of exendin-4 with a 2.4-hour half-life time, a proof of concept that is now foundational to the field (Cai et al., 2013). Second-generation agonists like liraglutide and semaglutide introduced fatty acid acylation to promote albumin binding, extending the time in circulation to 13 hours and 7 days, respectively (Knudsen and Lau, 2019). Dulaglutide achieved once-weekly dosing via an IgG4 Fc fusion, another well-established structural strategy. The field is currently moving toward unimolecular polypharmacology (Jimenez-Solem et al., 2010). Tirzepatide, a dual GLP-1R and GIP receptor (GIPR) agonist, has demonstrated superior efficacy in glycemic control and weight loss, a result now robustly supported by randomized head-to-head trials (Aronne et al., 2025; Frias et al., 2021). Regarding newer agents, evidence from phase 2 trials indicates that the triple agonist retatrutide (GLP-1R/GIPR/GCGR) can achieve unprecedented weight loss of up to 24.4% (Jastreboff et al., 2023). However, although the metabolic efficacy of these emerging agents is clear, their long-term cardiovascular safety profiles remain speculative until the conclusion of ongoing phase 3 trials (Abdrabou Abouelmagd et al., 2025). Similarly, oral formulations such as orforglipron show high potential for improving adherence, but their safety in humans remains under monitoring (Wharton et al., 2025).

Experimental evidence derived from the integrated study of the endogenous GLP-1R and GLP-1RAs indicates that this signaling axis mitigates oxidative stress through multiple pathways documented in preclinical studies (Ghosh et al., 2023). It is established that GLP-1R activation induces the stabilization of Nrf2, potentially via PKA or PKCδ, enabling its translocation to the nucleus (Fernandez-Millan et al., 2016; Kim et al., 2017). Once bound to the antioxidant response element (ARE), Nrf2 upregulates cytoprotective genes such as Heme Oxygenase-1 (HO-1) and NAD(P)H: Quinone Oxidoreductase 1 (NQO1) (Kim et al., 2017; Zhou et al., 2016). Furthermore, experimental evidence indicates that Epac-mediated inactivation of Src kinase inactivation inhibits the NADPH oxidase (NOX) system, thereby reducing ROS generation (Gianni et al., 2008; Mukai et al., 2011). Mitochondrial health is also revitalized by these agents. It is documented that GLP-1 treatment induces a significant increase in PGC-1α expression via the cAMP/PKA/CREB axis (Kang et al., 2006, 2015; Wu et al., 2022; Xie et al., 2021). This biogenic program upregulates mitochondrial transcription factor A (TFAM) and electron transport chain (ETC) proteins, thereby increasing oxygen consumption and ATP/ADP ratios (Hodson et al., 2014; Kang et al., 2015). While the biogenic effect is certain, the exact contribution of localized signalosomes to these mitochondrial improvements, compared to canonical plasma membrane signaling, remains unknown.

The ER is a central organelle for proinsulin folding, a capacity that is often overwhelmed in insulin-resistant states (Arunagiri et al., 2018). The evidence shows that GLP-1R signaling mitigates ER stress by upregulating the major chaperone BiP (GRP78), which keeps UPR sensors inactive (Cunha et al., 2009). By activating the PKA/CREB pathway, GLP-1RAs promote the expression of pro-survival genes such as Bcl-2 and JunB, thereby counteracting the pro-apoptotic signals of CHOP (Cunha et al., 2014, 2009). In hepatocytes, these mechanisms have been shown to enhance autophagy and the clearance of lipid droplets, which is critical for preventing the progression of MASH (Fang et al., 2020; Sharma et al., 2011). The cellular protection of β-cells and hepatocytes is well-supported by *in vitro* and animal data, anti-steatohepatitis effects are supported, while reversal of established fibrosis remains uncertain pending more definitive phase 3 outcomes (Loomba et al., 2023; Newsome et al., 2021).

Despite ongoing clinical characterization of these structural modifications, the evidence is clear that these intracellular cascades translate into robust systemic improvements that have redefined the standard of care (Abu-Nejim and Becker, 2025). The translation of molecular signaling into systemic benefits represents a paradigm shift in metabolic management. The robust reduction in HbA1c (0.5% to 1.5%) is anchored in the PKA-mediated sensitization of the β-cell, a mechanism that remains glucose-dependent and thus carries a low risk of hypoglycemia (Alharbi, 2025). Furthermore, a 12%- 15% reduction in major adverse cardiovascular events (MACE) (Lee et al., 2025) is supported by high-quality evidence from trials such as LEADER and SUSTAIN-6 (Marso et al., 2017). This benefit is likely mediated by the Nrf2-dependent reduction of endothelial ROS and the stabilization of vascular barriers.

The alignment of these systemic clinical outcomes with the preservation of intracellular structures underscores a profound integration of endocrine control and organellar maintenance. This convergence implies that the biological actions of GLP-1 receptor agonists are not merely the result of diffuse signaling events, but instead arise from a highly regulated cellular architecture. Such a framework suggests that the GLP-1 receptor coordinates its diverse metabolic functions by organizing into specialized platforms that directly engage with the Mitochondria-ER interface.

## 4 MERCS as a structural signaling platform to GLP-1 receptor

Contemporary understanding of the GLP-1R has transitioned from a model of linear, diffuse signaling at the plasma membrane to a sophisticated architecture of spatiotemporally organized nanodomains known as signalosomes (Austin and Tomas, 2026). Central to this organization are the MERCS, which serve as dynamic structural signaling platforms where the GLP-1R coordinates essential metabolic functions, including Ca^2+^ flux, lipid transport, and bioenergetic restoration. This integration of incretin signaling at the ER-mitochondria interface represents a fundamental and conserved mode of action across multiple metabolic tissues, including pancreatic β-cells, vascular smooth muscle, and the central nervous system (Morales et al., 2014; Spezani and Mandarim-de-Lacerda, 2026; Torres et al., 2016).

The GLP-1R achieves signaling specificity through the formation of Receptor-Associated Independent Nanodomains (RAiNs), which are concentrated hubs of approximately 60 nm in size designed for cAMP accumulation (Anton et al., 2022; Buenaventura et al., 2019). Evidence established that these nanodomains ensure high signal fidelity even at low ligand concentrations, preventing signal dissipation into the bulk cytosol (Anton et al., 2022). While these pathways are well established, it remains unclear whether RAiNs protect cAMP from rapid hydrolysis by phosphodiesterases, although the precise temporal regulation of these signalosomes in a living human environment remains a subject of ongoing investigation. The spatial compartmentalization provided by MERCS is vital for this precision, as it allows internalized, endosomal GLP-1Rs to engage with specific local effectors without interfering with other cytosolic pathways.

**Figure 2:**
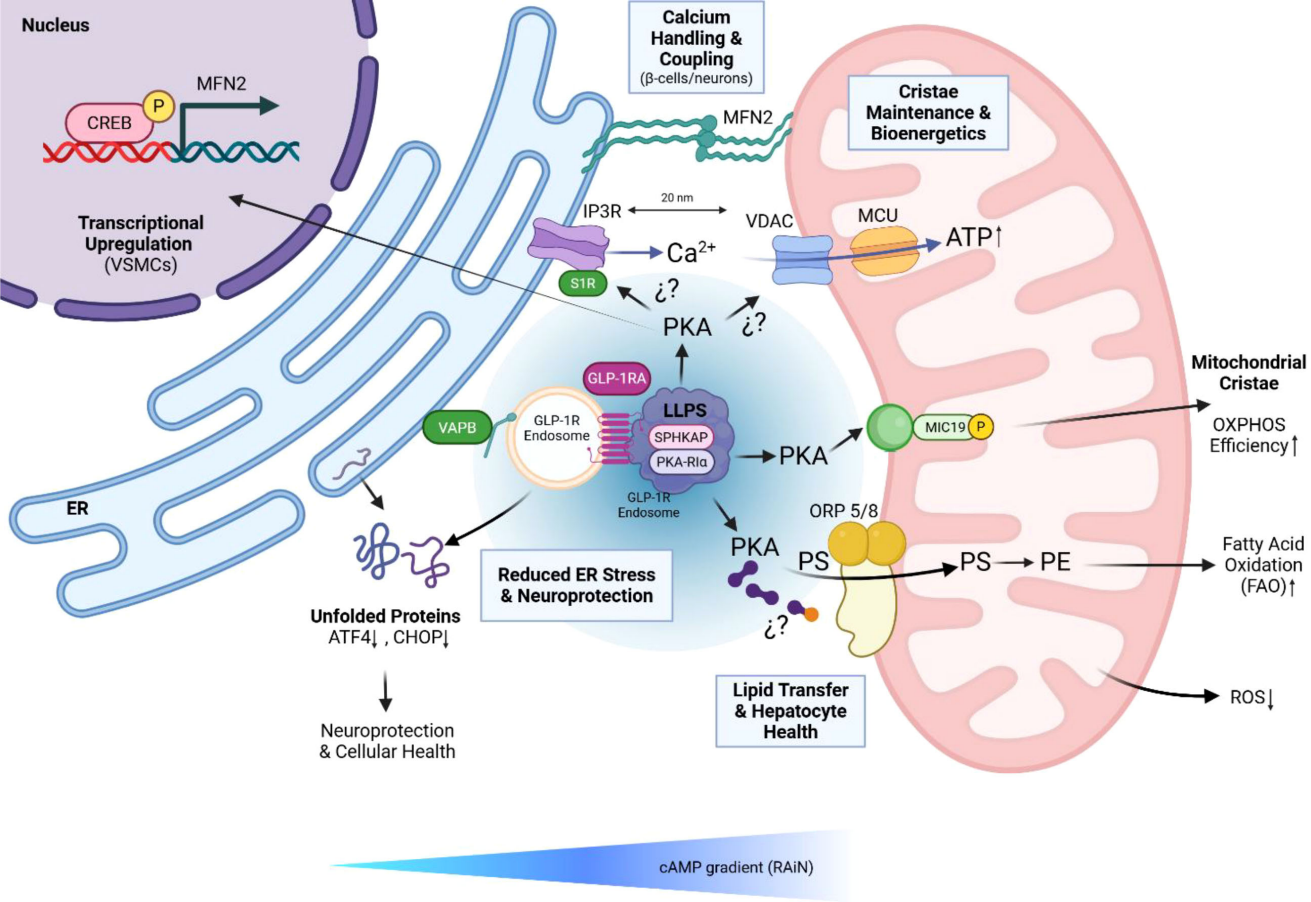
The pleiotropic role of the GLP-1R signalosome at Mitochondria-ER Contact Sites. This schematic illustrates the multifaceted restorative actions of GLP-1 Receptor Agonists (GLP-1RA) on MERCS function. The central “command center” is the endosomal GLP-1R signalosome, tethered to the endoplasmic reticulum (ER) by VAPB, where it forms a biomolecular condensate via liquid-liquid phase separation (LLPS) with the A-kinase anchoring protein SPHKAP and the PKA regulatory subunit PKA-RIα. This localized hub orchestrates diverse downstream pathways: Transcriptional Upregulation (Top Left): In Vascular Smooth Muscle Cells (VSMCs), PKA phosphorylates the nuclear transcription factor CREB, leading to the upregulation of the MFN2 gene and subsequent increase in MFN2 protein tethers, reinforcing ER-mitochondrial coupling. Calcium Handling & Coupling (Top Center): In β-cells and neurons, signalosome activity stabilizes the IP3R-VDAC/MCU complex, facilitating efficient Ca^2+^ transfer from the ER to the mitochondria and promoting ATP production. The exact direct targets of PKA phosphorylation in this complex (e.g., S1R) remain under investigation, as indicated by dashed arrows. Cristae Maintenance & Bioenergetics (Right): PKA phosphorylates specific targets such as MIC19 on the inner mitochondrial membrane. This promotes cristae organization and enhances oxidative phosphorylation (OXPHOS) efficiency. Lipid Transfer & Hepatocyte Health (Bottom Right): Proposed mechanisms suggest PKA signaling may modulate the activity of lipid transfer proteins like ORP5/8, facilitating the essential transport of phosphatidylserine (PS) to the mitochondria for conversion to phosphatidylethanolamine (PE), which is crucial for resolving hepatic steatosis. Reduced ER Stress & Neuroprotection (Bottom Left): The overall restoration of MERCS function by GLP-1RAs leads to a decrease in unfolded proteins and the downregulation of ER stress markers like ATF4 and CHOP, contributing to neuroprotection and cellular health. All these events are contained within a localized cAMP gradient, termed a Receptor-Associated Independent Nanodomain (RAiN). Created with BioRender.com.

This signaling architecture is supported by a core set of molecular components. The integral ER protein VAPB acts as a primary structural tether, recruiting endosomal GLP-1Rs to the MERCS upon agonist stimulation (Austin et al., 2025). Within this hub, the A-kinase anchoring protein SPHKAP serves as a scaffold that organizes biomolecular condensates of PKA regulatory subunits via liquid-liquid phase separation (LLPS) (Austin et al., 2025) (**Figure 2**). These condensates create privileged microenvironments for the localized activation of PKA, restricting its activity to the mitochondrial vicinity to ensure phosphorylation of specific targets such as MIC19 (Folkmanaite and Zaccolo, 2025; Zhang et al., 2020). MIC19 phosphorylation regulates cristae junctions and lipid precursor transfer across the MERCS, a process essential for cardiolipin biosynthesis and the maintenance of mitochondrial respiratory complexes (Dong et al., 2024; Friedman et al., 2015). Disruption of this hub, via silencing of VAPB or SPHKAP, significantly blunts the ability of the receptor to potentiate insulin secretion and protect β-cells from apoptosis (Austin et al., 2025).

The capacity of GLP-1R to enhance ER-mitochondria coupling mechanism is extended to the vascular system. In vascular smooth muscle cells (VSMCs), GLP-1 treatment induces a metabolic shift characterized by increased mitochondrial oxidation and bioenergetic efficiency (Morales et al., 2014; Torres et al., 2016). Unlike the rapid recruitment mechanism seen in β-cells, GLP-1 promotes organelle coupling in VSMCs through the PKA-dependent transcriptional up-regulation of MFN2. Within three hours of treatment, MFN2 levels approximately double, strengthening the physical and functional communication between the ER and mitochondria. This enhanced coupling facilitates a more efficient transfer of Ca^2+^ from ER release sites (IP3Rs) to the mitochondrial matrix, activating Krebs cycle dehydrogenases and elevating cellular ATP levels. Furthermore, GLP-1 reduces the inactivating phosphorylation of PDH, effectively redirecting glucose metabolism away from anaerobic glycolysis toward more efficient mitochondrial oxidation (Morales et al., 2014; Torres et al., 2016).

This signaling platform at MERCS also serves as a critical regulator of mitochondrial quality control. GLP-1R activation increases the recruitment of the fission protein Drp1 to mitochondria and enhances mitophagy, thereby removing damaged organelles and protecting tissues from oxidative stress (Chen et al., 2024; Torres et al., 2016). In the central nervous system, these signalosomes have been linked to neuroprotective effects in Alzheimer’s and Parkinson’s diseases by reducing neurotoxic ER stress (Reich and Holscher, 2022). Similarly, in metabolic dysfunction-associated steatotic liver disease (MASLD), GLP-1R agonists resolve hepatic steatosis by promoting healthy MERCS dynamics, thereby optimizing fatty acid oxidation (Lee et al., 2012) (**Figure 2**). The identification of MERCS as a structural signaling platform marks a shift toward a contactomics approach in incretin research, providing a framework to dissect how organelle miscommunication drives the progression of obesity and T2DM. Future therapeutic strategies may rely on the ability of “biased” agonists to favor receptor internalization and prolonged residence in MERCS-coupled endosomes, thereby offering a refined restoration of mitochondrial health and improved systemic metabolism.

## 5 Discussion: perspective and open questions

While the identification of RAiNs (Anton et al., 2022) and MERCS-associated signalosomes (Austin et al., 2025) defines the current frontier of incretin research, several critical gaps remain. A paramount question is whether this signaling architecture operates effectively within aging populations or during chronic metabolic collapse. In advanced disease, persistent organelle uncoupling and progressive depletion of structural tethers, particularly MFN2 (Sebastian et al., 2012) and VAPB (Austin et al., 2025), may create a structural barrier that impedes efficient assembly of GLP-1R signalosomes. Furthermore, the aging process entails a baseline decline in mitochondrial communication and the accumulation of senescent markers (Janikiewicz et al., 2018), which likely compromise the biophysical environment required for LLPS and biomolecular condensate nucleation in β-cells (Austin et al., 2025). Determining whether GLP-1 receptor agonists can reverse these established structural deficits (Huang et al., 2025) or only delay further decay is essential for de-risking clinical translation and defining the therapeutic window for these agents in late-stage pathologies.

The role of calcium signaling at these interfaces represents another pressing open question. Unlike the well-characterized cAMP axis, direct evidence describing how GLP-1 receptor agonists modulate Ca^2+^ flux within signalosomes remains fragmentary. It is still unknown whether PKA-mediated phosphorylation within these hubs directly targets IP3Rs isoforms or the mtCU complex to fine-tune mitochondrial energy uptake. Furthermore, the therapeutic window for contact-site modulation poses a significant challenge. Because MERCS are kinetic assemblies, pharmacological interventions must avoid hyper-stabilization that could precipitate mitochondrial Ca^2+^ overload or misroute vital second messengers (Thoudam et al., 2023). Determining the optimal zone for organellar proximity will require dose- and kinetics-aware strategies guided by real-time biomarkers. Establishing a systematic incretin contactome atlas using super-resolution imaging and single-cell spatial omics will be vital to bridging these gaps and informing the design of next-generation therapies capable of precisely restoring intracellular structural and functional integrity (Austin et al., 2025).

A further layer of complexity is that MERCS remodeling is unlikely to follow a uniform, linear trajectory across tissues and disease stages. Evidence from metabolic organs suggests a potential biphasic behavior, early contact enrichment and heightened flux followed by late-stage decoupling (Theurey and Rieusset, 2017), raising the possibility that GLP-1R agonists may exert qualitatively different effects depending on whether the limiting factor is excessive Ca^2+^ transfer versus structural collapse of tethering and scaffolding networks. In this context, incretin resistance might partially reflect a failure of signal compartmentalization: even when bulk cAMP rises, local RAiN/MERCS nanodomains may be unable to sustain effective signaling (Anton et al., 2022) due to altered receptor trafficking (reduced endosomal residency near contacts) and/or increased PDE-driven cAMP erosion within microcompartments (Austin et al., 2025). These considerations motivate a shift from purely pathway-centric models to spatiotemporally quantitative frameworks that track receptor dwell time, contact-site turnover, and local second-messenger dynamics in real time. Practically, the field would benefit from biomarkers that report contact integrity and function (e.g., ER–mitochondria proximity indices, tether/scaffold abundance states, localized PKA/EPAC activity reporters, and Ca^2+^ microdomain readouts) integrated with single-cell spatial profiling across age and disease severity (Austin et al., 2025; Manchanda et al., 2024). Such an atlas would not only clarify the therapeutic window for contact-site modulation but also enable patient stratification and rational combinations aimed at restoring—not merely amplifying—intracellular signaling fidelity.

## Glossary

AC: Adenylate cyclase
AMFR: Autocrine motility factor receptor
AMPK: AMP-activated protein kinase
ARE: Antioxidant response element
ATF4: Activating transcription factor 4
ATF6: Activating transcription factor 6
cAMP: Cyclic adenosine monophosphate
CHOP: C/EBP homologous protein
cryo-EM: Cryogenic electron microscopy
DAG: Diacylglycerol
DPP-4: Dipeptidyl peptidase-4
ECD: Extracellular domain
Epac2: Exchange protein directly activated by cAMP 2
ER: Endoplasmic reticulum
ETC: Electron Transport Chain
FFAs: Free fatty acids
GLP-1: Glucagon-like peptide-1
GLP-1R: Glucagon-like peptide-1 receptor
GLP-1RA: Glucagon-like peptide-1 receptor agonist
GPCR: G protein-coupled receptor
GRP75: Glucose-regulated protein 75
GSIS: Glucose-stimulated insulin secretion
IP3: Inositol 1,4,5-trisphosphate
IP3R: Inositol 1,4,5-trisphosphate receptor
LLPS: Liquid-liquid phase separation
MASH: Metabolic dysfunction-associated steatohepatitis
mtCU: Mitochondrial calcium uniporter complex
MCS: Membrane contact site
MERCS: Mitochondria-ER contact sites
MFN2: Mitofusin 2
MICOS: Mitochondrial contact site and cristae organizing system
mPTP: Mitochondrial permeability transition pore
NADH: Nicotinamide adenine dinucleotide (reduced form)
Nrf2: Nuclear factor erythroid 2-related factor 2
OMM: Outer mitochondrial membrane
ORP: Oxysterol-binding protein-related protein
OXPHOS: Oxidative phosphorylation
PE: Phosphatidylethanolamine
PISD: Phosphatidylserine decarboxylase
PKA: Protein kinase A
PS: Phosphatidylserine
RAiNs: Receptor-associated independent nanodomains
ROS: Reactive oxygen species
S1R: Sigma-1 receptor
T2DM: Type 2 diabetes mellitus
TBK1: TANK-binding kinase 1
TMD: Transmembrane domain
UPR: Unfolded protein response
VAPB: Vesicle-associated membrane protein-associated protein B
VDAC: Voltage-dependent anion channel
